# Neurophysiological markers of cognitive workload under altered gravity conditions using a gamified dual-task paradigm

**DOI:** 10.1038/s41598-025-34426-0

**Published:** 2026-01-09

**Authors:** Constance Badalì, Petra Wollseiffen, Lennart Puck, Timo Klein, Stefan Schneider

**Affiliations:** 1 Institute of Movement and Neurosciences, German Sport University Cologne, Am Sportpark Müngersdorf 6, 50933 Cologne, Germany; 2 Department of Laboratory Medicine, Division of Clinical Physiology, Karolinska Institutet, Stockholm, Sweden; 3 Centre for Health and Integrative Physiology in Space (CHIPS), German Sport University Cologne, Cologne, Germany; 4 Robotics and Automation Section, European Space Agency, Noordwijk, Netherlands; 5 Institute of Sports Science, University of Rostock, Rostock, Germany; 6 Centre for Transdisciplinary Neurosciences Rostock, University of Rostock, Rostock, Germany

**Keywords:** Weightlessness, Hypergravity, Parabolic flight, Behavioural and neuronal parameters, ERP, EEG, Neuroscience, Psychology, Psychology

## Abstract

Astronauts must maintain optimal cognitive function to complete critical tasks in demanding environments. However, stress and workload can impair executive functions and decision-making, which may ultimately affect mission success. To evaluate workload management the study investigated behavioural data (Reaction Time and Error Rate), as well as neurophysiological parameters (event-related potentials and electrocortical activity) during a continuous primary task combined with an auditory oddball paradigm as a secondary task. Data were collected across Earth gravity, hypergravity (1.8 G), and weightlessness in 25 consecutive parabolas through parabolic flights. Electrocortical activity and performance on the primary task showed no differences between gravity levels. However, the response to target stimuli in the secondary task in microgravity showed a significant increase in the error rate compared to hypergravity and normal gravity. Electrophysiological data showed a pronounced N100-P200 complex, indicating perception-related processing of the oddball paradigm sound. The typical fronto-central N200 component was triggered by both sounds of the oddball paradigm, although no differences were observed between the gravity levels. The absence of a P300 component which is an indicator of higher cognitive processing, suggests that most cognitive resources were devoted to the primary task, reducing the discrimination of the auditory stimuli. Furthermore, the presence of the N200 component, interpreted as the mismatch negativity (MMN), indicates automatic neural responses to auditory deviations that are independent of cognitive load. These findings emphasise that cognitive bottlenecks can arise easily in demanding operational environments, where cognitive resources are limited. Effective task prioritisation and training-based automation are therefore essential for sustaining performance during human spaceflight. Given the substantial variability across individuals, personalised workload-management strategies may be crucial for ensuring focus, timely decision-making, and mission safety. Further research is needed to explore cognitive workload management in such demanding environments.

## Introduction

Since space missions regularly require astronauts to perform complex, critical tasks in challenging environments, maintaining cognitive performance is of great importance. In situations of increased stress, cognitive processes of executive functions and decision-making can be negatively affected. Such impairment presents a notable risk to missions where the ability to perform complex motor tasks safely and efficiently is critical. The training and development of routines for these procedures are important for mission success, but possibly allow astronauts to free up or reallocate cognitive resources to adequately resulting in enhanced coping of unexpected tasks. The relevance of monitoring and maintaining cognitive performance in space is a crucial point of ESA’s strategic roadmaps^13^ and of major importance when designing and applying countermeasures to increase mission success and safety. To optimise interventions, the combination of physiological and psychological information is needed. This includes reaction time and error rates to monitor neuro-psychological status during a mission^16^ and provide significant data regarding the physical and mental health of the crew^10^. While cognitive performance and its underlying neurophysiological mechanisms are known to be critical for successful spaceflight, the integration of physical and psychological data remains poorly understood. This gap limits our ability to fully assess and support astronaut performance and well-being in extreme environments.

In the last decade, several studies have investigated human behaviour and neurocognitive performance in microgravity, e.g. during space flights or experimental parabolic flights^26,27^. Despite anecdotal reports of astronauts and cosmonauts, there seems to be a positive impact of weightlessness on cortical processing^42,43^. These results are in line with in vitro data from^35^ reporting an increased excitability of neurons under microgravity^35^. Summarizing in vivo and in vitro experiments^25^, suggest a reduced membrane viscosity caused by increased lateral pressure on neurons in microgravity. This leads to a reduction in the open-state probability of ion channels, which results finally in a slightly depolarized resting membrane potential of the cell^35^. Studies already show that cells under external pressure tend to have reduced neuronal excitability^25^. It is therefore assumed, that the well-reported increase in blood volume to the brain during microgravity increases intracranial pressure in the brain and might be the cause for this cascade of physiological changes.

The human brain’s ability to perform cognitive tasks depends on whether it can efficiently and effectively use the necessary and available brain resources for the tasks at hand. It has been demonstrated that the utilisation of available brain resources is dependent on the challenge presented by the tasks to be completed^20,22^. The term ”cognitive workload” can be used to describe the concept of brain resource utilisation^6,7,39^. A possible neurophysiological representation of this can be observed in the amplitude and latency of specific event-related potentials (ERPs)^17^, i.e., the early components, such as the N100-P200 component, for stimulus perception and sensation, as well as later potentials, such as the P300, for stimulus evaluation and interpretation. On Earth, studies revealed a correlation between the amplitude of ERPs with the amount of attentional resources allocated to a given task^28^. Beyond conditions on Earth, recent studies show that ERPs are also proving useful in space research for investigating cognitive performance^4,5,11,24^. A study shows higher amplitudes of ERPs elicited by a discrete primary and secondary task in hypergravity without differences in behavioural data^4^. This is also true for the cognitive performance data under partial gravity (Moon/Mars) and for differences in ERP amplitude and latency, which could lead to the suggestion that there is a threshold for the influence of lower gravity on neurocognitive performance^5^.

All these results were obtained using the same experimental design, with a dual-task consisting of two discrete components. However, in real scenarios, they can differ in nature and can therefore affect cognitive resources allocation. Accordingly, a continuous navigation task (Pac-Man) was selected as the primary task with a non-continuous classical auditory oddball paradigm, as it provides a high level of intrinsic motivation (gamification). Given the game-like and more challenging character of the continuous primary task, it is hypothesized that performance under 0 G is increased compared to 1 G and 1.8 G and that this is simultaneously mirrored by a reduction of the elicited ERP waves during the experiment. The aim of this experiment is to investigate the effects of different gravity levels on a dual-task assignment and to gain a fundamental understanding of the distribution of cognitive resources when combining continuous and discrete structures. In the long term, this may be helpful in developing suitable coping strategies on Earth for stressful situations, as well as for task distribution and task design in spaceflight.

## Material and methods

### Participants and procedure

Parabolic flights take place on board the A310 ZeroG from Merignac International Airport in Bordeaux (F), led by the European Space Agency (ESA) and the German Aerospace Centre (DLR). Each parabola consists of four phases, characterised by shifts in gravity from Earth gravity to hypergravity, microgravity, back to hypergravity and returning to Earth gravity. Every campaign includes three flight days, with 30 experimental parabolas per day. Over three campaigns conducted from September 2023 to June 2024, data were collected from 18 participants (12 males, 6 females) who executed the experiment in normal gravity (1 G), hypergravity (1.8 G) and microgravity (0 G).

All participants were healthy and reported no history of neurological, psychiatric or cognitive disorders. They were all right-handed and received identical instructions. All participants went through the experimental protocol 24 hours before the start as a familiarisation process. The experimental design was strictly intra-individual, with each participant acting as their own control. This minimised the potential influence of factors such as age, gender, and task proficiency on the observed behavioural and electrophysiological differences between gravity conditions.

**Fig. 1:**
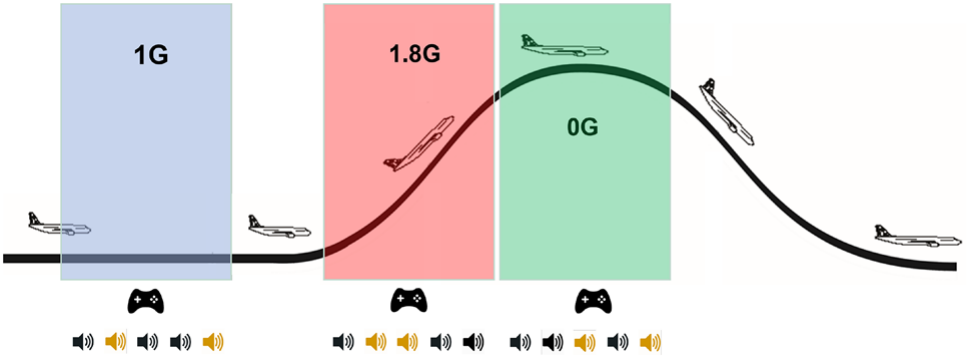
The experiment was conducted during the first three phases of a parabolic flight manoeuvre, starting with Earth gravity (1 G), followed by hypergravity (1.8 G) and weightlessness (0 G). The participants had to solve a dual-task (SpaceMan), which consisted of playing a continuous navigation task (Pac-Man) as a primary task and an auditory oddball paradigm as a secondary task. This was done during 25 consecutive parabolas.

All participants and investigators underwent clinical examinations and provided informed consent. The study’s experimental design was approved by the Research Ethics Committee of the German Sport University Cologne and the University of Caen following the Declaration of Helsinki. For a graphical representation, see Fig. 1.

## Experiment

The neurocognitive task consisted of a continuous navigation task (Pac-Man) and a classical auditory oddball task. Pac-Man is a universally recognised and stimulating game, which holds participants’ attention and encourages their best efforts, while still simulating the cognitive demands of mission-critical operations. Given its dynamic and goal-oriented character, it facilitates sustained cognitive effort throughout the experiment and counteracts fatigue across repeated parabolas.

The term *SpaceMan* refers to the modified version of the classic Pac-Man game used in this study to evaluate cognitive performance in different gravitational environments. It combines the name ’Pac-Man’ with the experimental context of spaceflight. Pac-Man was played using the arrow keys with the right hand.

While playing Pac-Man, the secondary task of this dual-task was a classical auditory oddball paradigm, where participants heard randomised tones through wired noise-cancelling headphones (BOSE QuietComfort 20). These tones included either target tones (high tones) or standard tones (low tones), which were both 80 ms long. The low-pitched tones made up 70% of all tones and had to be ignored, while the high-pitched tones, which made up 30% of all tones, prompted participants to quickly press the space bar with the left hand on a keyboard on their lap. The interstimulus interval was between 1.05 and 1.1 s, as the maximum allowed reaction time of the participants was set to 1s. Additionally, a small randomised offset of 0.05 to 0.1s was added before the next stimulus, so no distinct pattern was to emerge.

The auditory oddball paradigm used in this study has previously been validated and shown to elicit a reasonable brain response to different tones^4,5,41^.

**Fig. 2:**
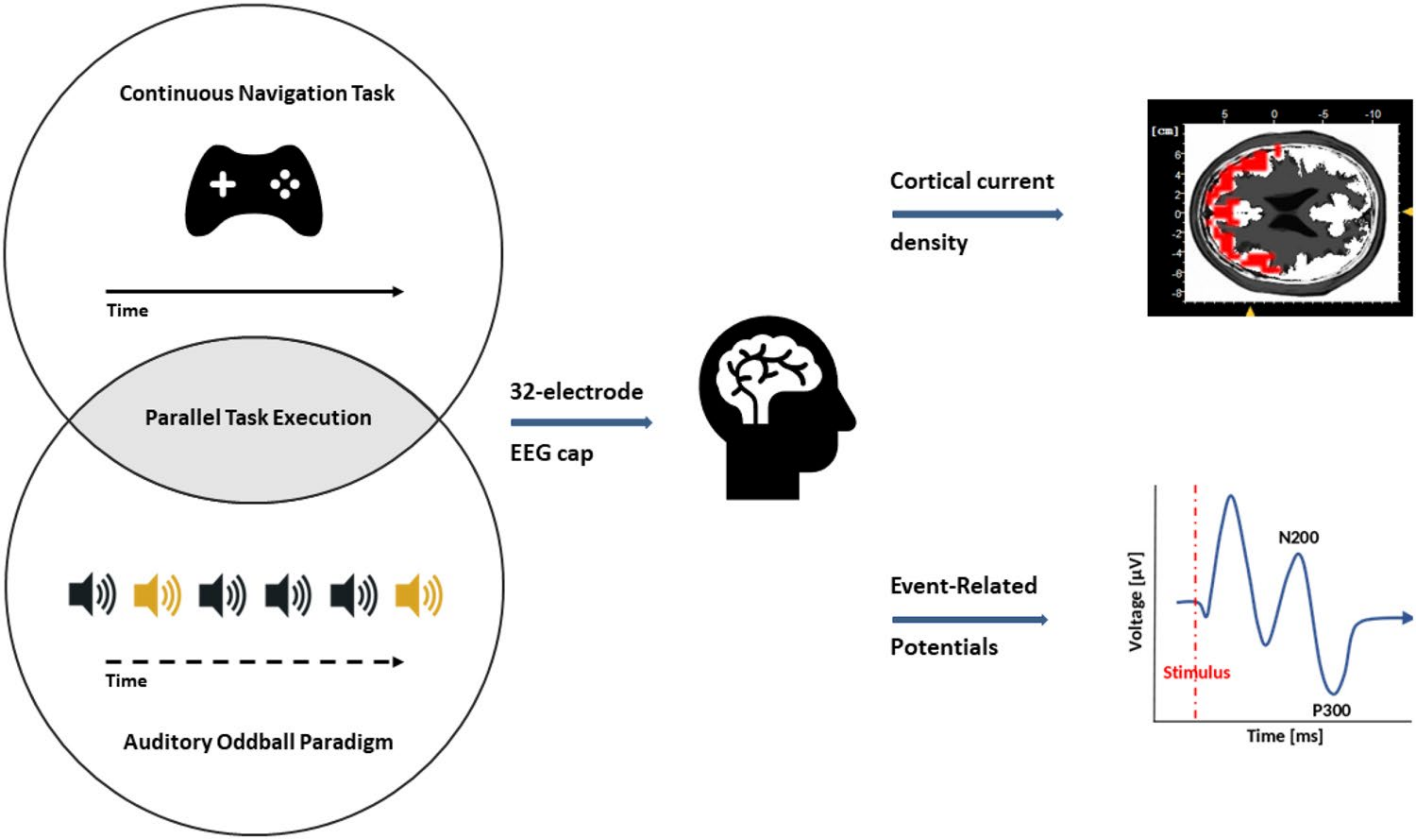
The neurocognitive task involved a continuous navigation task in combination with a classical auditory oddball paradigm (SpaceMan), which participants had to perform simultaneously during 1 G, 1.8 G and 0 G phases of a parabolic flight. This task was repeated in 25 consecutive parabolas for each of the specified gravity levels on a single flight day.

Each trial lasted for 18 seconds, allowing it to integrate into the 1.8G and 0G phases of the flight. Each participant performed 25 parabolas. A schematic representation of the experiment is shown in Fig. 2.

Participants were instructed to prioritise the Pac-Man task, aiming for a high score and to consider the sounds of the oddball paradigm as secondary. In order to minimise potential learning and habituation effects during the experiment, the initial position of Pac-Man and the movement patterns of the ghosts were randomized at the onset of each round, thereby ensuring that no predictable sequences occurred. All participants received Scopolamine (anti-nausea medication) right before the flight. The dose of the medication was calculated individually by the flight physicians, taking into account the body weight and experience (novice or experienced flyer) of each participant. Previous studies have shown that standard doses of scopolamine for motion sickness have no significant effect on attention or electrophysiological measurements^8,43^. The study’s within-subject design ensures any such effect, if present, would be consistent across all gravity conditions and therefore unlikely to explain the reported differences. Throughout the flight, the participants were sitting and safely secured to an aircraft seat, avoiding uncontrolled flying and loss of orientation, but allowing the participants to experience the sensation of microgravity. During the whole flight, the participants were observed by an operator to avoid external disturbances and to provide reassurance to the participants during the phases of altered gravity.

## EEG data collection

Each participant wore a 32-channel EEG cap (actiCap-32Ch, Brain Products GmbH, Munich, Germany) that was custom-fitted to their unique head size and arranged according to the classic 10–20 configuration. To ensure a consistent data acquisition, each electrode was referenced to a reference electrode positioned within the triangle formed by FP1, FP2, and Fz, with the ground electrode placed adjacent to it. To facilitate optimal signal transmission, all electrodes were filled with Electro-Gel™ (Electro-Cap International, USA). To maintain impedance levels below ten kilo-Ohms [$\,\text {k}\Omega$] throughout the flight, the electrodes were regularly refilled with gel. Before being stored, EEG data were subjected to analogue-to-digital conversion and amplification using Brain Vision amplifier and RecView software (Brain Products GmbH, Munich, Germany).

## Data analysis

EEG data preprocessing and analysis were performed using Brain Vision Analyzer 2.2 (Brain Products GmbH, Munich, Germany). Data that provides information about the participants’ performance, such as error rate and reaction time, was analysed using self-created scripts in Python 3 (Python Software Foundation, https://www.python.org/^37^) in Jupyter Notebooks.

## EEG analysis

After filtering the EEG signals with low-pass and high-pass techniques, a frequency range from 0.5 to 30 Hz was retained for the analyses (time constant of 0.318 s and 48 dB/octave). Individual channels exceeding $10\,\text {k}\Omega$ of impedance were interpolated using splines (order: 4, maximum degree of Legendre polynomials: 10, standard lambda: 1E-05). To detect and remove blinks and horizontal eye movements, independent component analysis was implemented after a visual inspection of the dataset. *Current cortical density* After the initial segmentation into 1 G and the respective gravity level, followed by division into 4 s intervals, an automatic artefact rejection was conducted (gradient < 50 $\,\mu \text {V}$; max/min amplitude $-200$ to $200\,\mu \text {V}$; lowest allowed activity in intervals 0.5$\,\mu \text {V}$). As scalp potentials vary in volume, based on reference location, the data was converted into reference-free current source density (CSD) maps for every participant (order of splines: 4; maximum degree of Legendre polynomials: 10; lambda 1e-5). The CSD consists of the voltage values of individual electrodes in addition to the current source density recorded at these electrodes. Using the integrated LORETA module in the Brain Vision Analyzer^18,33^, cortical current densities in the frontal, parietal, and occipital lobes and the region supplied by the middle cerebral artery (MCA - Middle Frontal Gyrus LR, Precentral Gyrus LR, Middle Temporal Gyrus LR, Inferior Frontal Gyrus LR, Superior Temporal Gyrus LR, Postcentral Gyrus LR, Inferior Parietal Lobule LR) were determined across each 4-second recording interval. Cortical current density is defined as the electric current triggered by neural activity per unit area of cross-section. In general, the unit is microvolts per square millimetre (electrical current in a 2-dimensional area) but in a voxel-based analysis, this value needs to be squared so that the unit is squared microvolts per millimetre to the power of 4.

*Event-related potentials*

ERPs not only reflect how sensory information is perceived and processed but also indicate higher cognitive processes^12^. In the field of behavioural control processes, which encounters stimuli evaluation, selective attention, and conscious perception, two ERP components, N200 and P300, are important^19,34^. The appearance of ERPs is dependent on the occurrence of a specific stimulus, whether it be sensory, visual, or auditory, unlike that of spontaneous EEG. Due to that time-specific character, the data set was segmented based on the relevant stimulus, which was the appearance of target and standard tones of the oddball paradigm from $-200$ to 800 ms. Following that segmentation, data were corrected for artefacts (gradient<50 $\,\mu \text {V}$; max/min amplitude $-250$ to 250 $\,\mu \text {V}$; lowest allowed activity in intervals 0.5 $\,\mu \text {V}$) and baseline corrected ($-200$ to 0 ms). For the whole experiment, there were on average 84 stimuli for the target sound, and around 360 stimuli for the standard sound presented to each participant. Subsequently, the averaged ERPs for all participants under both, the 1G and partial gravity conditions and across all electrodes were computed, and a reference-free CSD map was obtained from all ERP waveforms using spline interpolation (order of splines: 4, maximum degree of Legendre polynomials: 10, default lambda: 1e-5). To determine the temporal occurrence (latency) and magnitude of the ERP amplitude, a peak algorithm was applied to the data sets. The time window for determining the parameters was 90 - 200 ms for the first negative wave. 100–250 ms for the consecutive negative wave and 150–300 ms for the negative wave over the fronto-central part.

## Statistics

Statistics were performed using self-written scripts in R (2022.12.0, Posit Software, PBC). Statistical analyses were performed using the **rstatix** package^23^, and data were visualised with **ggplot2**^40^. All data sets were tested for normality using the Shapiro-Wilk test before further statistical calculations. Further statistical evaluation was carried out with two-way repeated measures analysis of variance (RM ANOVA) or the corresponding non-parametric procedure, depending on the outcome of the Shapiro-Wilk test. Comparisons of reaction time (RT) were performed using either the Friedman test or a one-way repeated measures ANOVA with the within-group factors of *gravity* (1G/1.8G/0G). Comparison of the accuracy/percentage of errors (ER) was performed using either the Friedman test or a two-way repeated measures ANOVA with the within-group factors of *gravity* (1G/1.8G/0G) and *experiment* (target or standard sound of the oddball paradigm). Where appropriate, a post hoc analysis with Bonferroni correction was carried out. Additionally, as this study was conducted with a small sample size and the observation of outliers, the non-parametric version of a two-factorial repeated measures ANOVA, the ld.f2 function with Wald-type (WTS) of the nparLD package in R, was used^9,14,32^. This package refers to the results of the studies by^1^. Here, the relative treatment effects are defined concerning the distributions of the variables measured in the experiment. If this package provided a significant interaction effect of both factors, a Wilcoxon test was performed as a post-hoc test with Bonferroni correction. The neurophysiological parameters were analysed regarding their amplitude and latency in respect to the tones of the auditory Oddball paradigm.

If statistical significance was determined by the procedures, a post hoc test with Bonferroni correction was carried out. The level of significance was set to p < 0.05. Data in this manuscript are presented as mean and standard deviation.

## Results

**Fig. 3:**
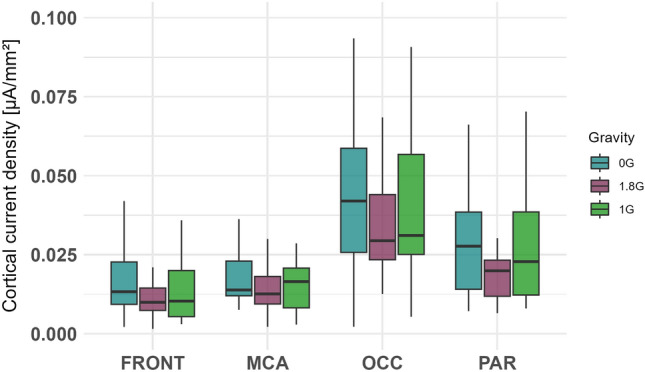
Cortical current density averaged over 25 parabolas for each gravity level in 0G, 1.8G and 1G. Displayed are means ± standard deviation. *FRONT* frontal lobe, *PAR* parietal lobe, *OCC* occipital lobe, *MCA* Brain region supplied by the middle cerebral artery.

**Fig. 4:**
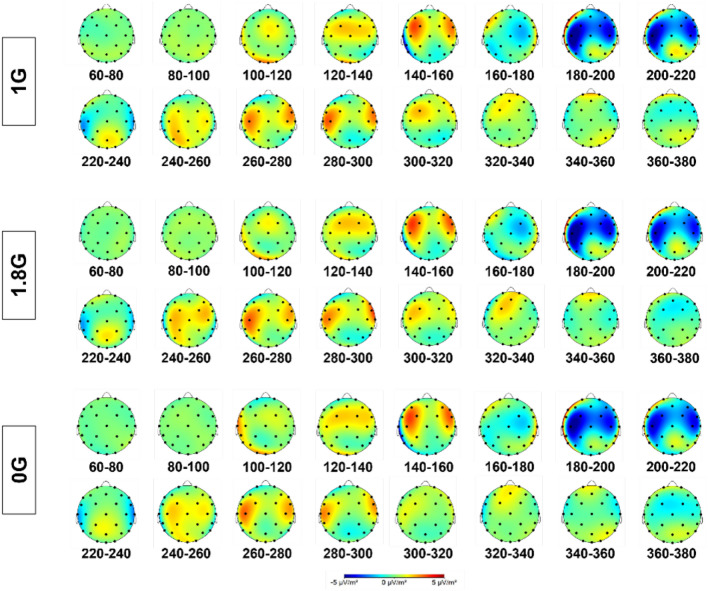
Topographical map in steps of 20 ms ranging from 60 ms – 380 ms after the occurrence of a standard sound in an auditory oddball paradigm over the scalp for 1G, 1.8G and 0G.

**Fig. 5:**
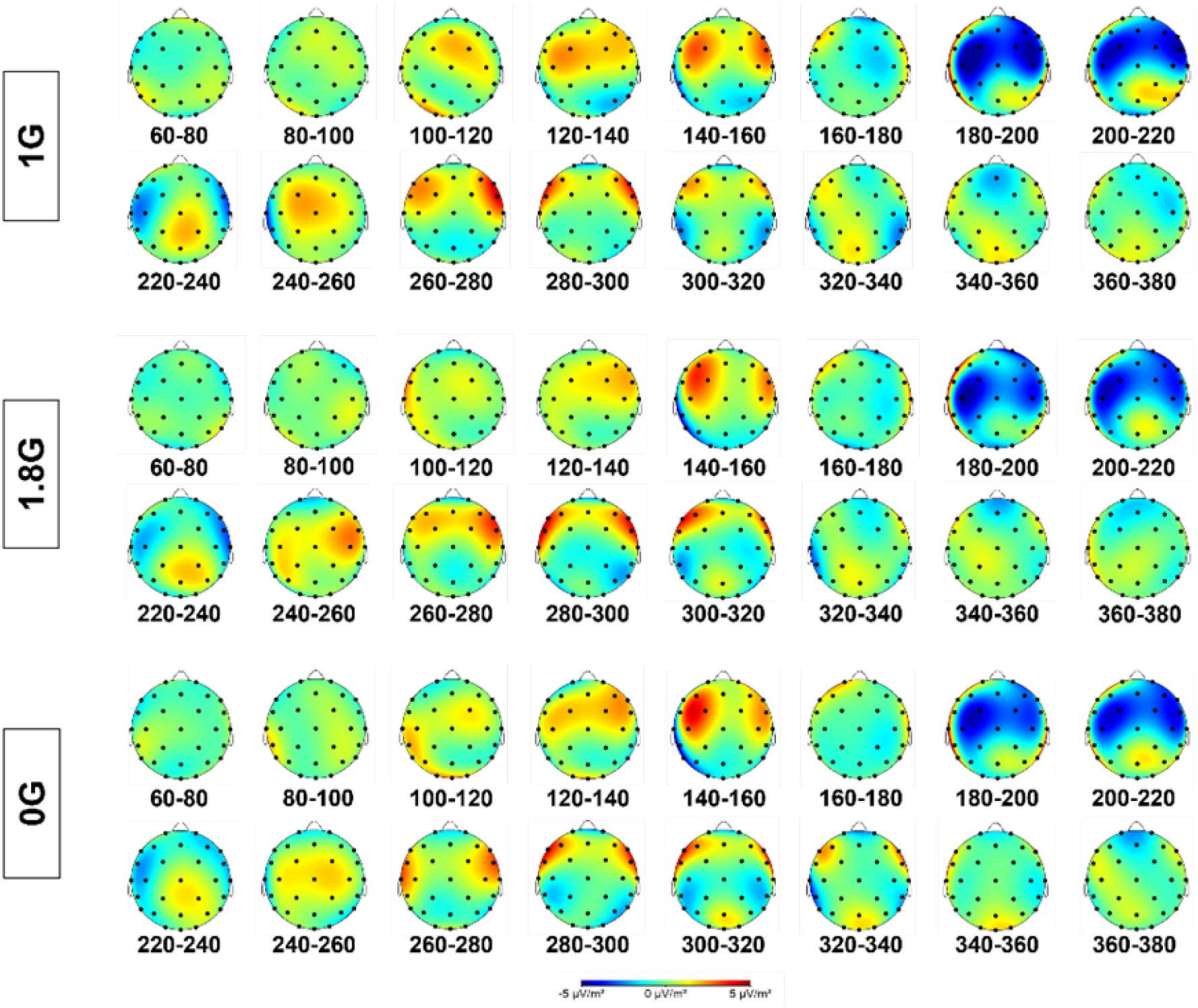
Topographical map in steps of 20 ms ranging from 60 ms – 380 ms after the occurrence of a target sound in an auditory oddball paradigm over the scalp for 1G, 1.8G and 0G.

The data for the electrocortical activity were not normally distributed and therefore the ld.f2 model with the Wald-type (WTS) was calculated and showed no significant interaction effect of the factors *ROI* and *gravity *(Statistic=4.665, p=.587, Figs. 3, 4, 5, Table 1).

**Table 1:** Cortical current density averaged over 25 parabolas for each gravity level. Displayed are means ± standard deviation. *FRONT* frontal lobe, *PAR* parietal lobe, *OCC* occipital lobe, *MCA* middle cerebral artery.

	Electrocortical Activity			
	FRONT	PAR	OCC	MCA
0 G	$0.016 \pm 0.011$	$0.034 \pm 0.0304$	$0.048 \pm 0.035$	$0.018 \pm 0.010$
1 G	$0.014 \pm 0.010$	$0.027 \pm 0.017$	$0.041 \pm 0.024$	$0.015 \pm 0.008$
1.8 G	$0.014 \pm 0.011$	$0.024 \pm 0.019$	$0.040 \pm 0.032$	$0.017 \pm 0.013$
Statistical test	Ld.f2 model with Wald test			
*p value*	n.s			

**Fig. 6:**
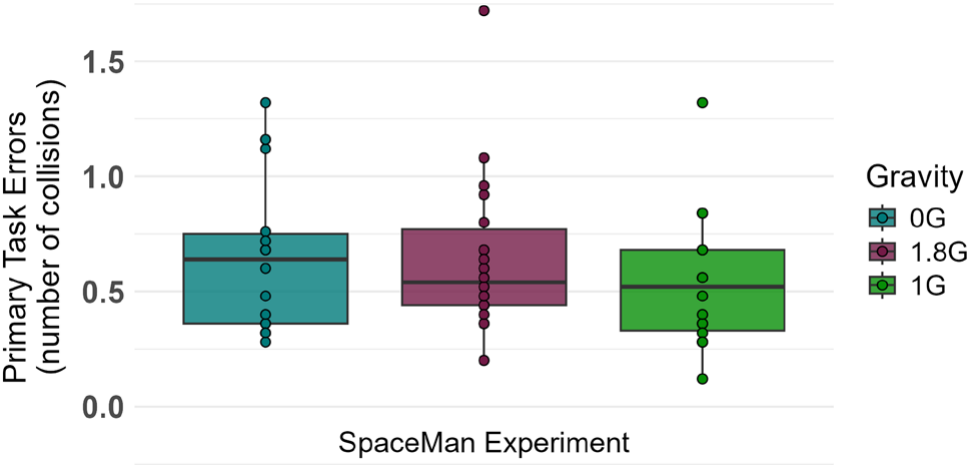
Performance of the primary task in 0G, 1.8G and 1G. To quantify the performance in the primary task, the average number of collisions between Pac-Man and the ghosts per round was calculated for each gravity level and used as an index of task efficiency.

To quantify the performance in the primary task, the average number of collisions between Pac-Man and the ghosts per round was calculated for each gravity level and used as an index of task efficiency. A Friedman test revealed no significant interaction effect (Fig. 6).

**Fig. 7:**
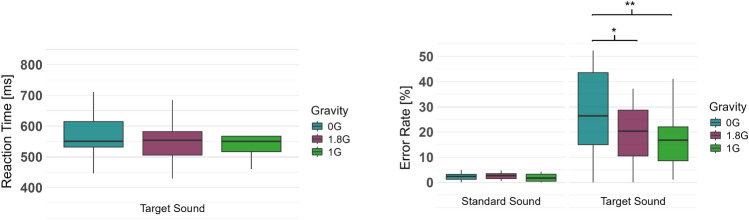
Reaction Time (left) on the target sound of the auditory oddball paradigm in 0G, 1.8G, and 1G, and Error Rate (right) for the standard and target sound in the same conditions. Displayed are means ± standard deviation.

The Friedman test regarding the participants’ reaction time on the target sounds of the oddball paradigm did not show a significant difference between the three gravity conditions ($\chi ^2$: 4.333, p=.115, Kendall W = 0.12, Fig. 7, Table 2).

The two-way repeated measures ANOVA for the participants’ accuracy, depicted as the error rate in percentage, showed a significant interaction effect between the different gravity levels and the processing of the target sound of the auditory oddball paradigm (F_(34,2)_=8.986, p=.003, $\eta ^2_G$ = 0.046, 95% CI = [0.015, 0.234], Fig. 7, Table 2). Post-hoc pairwise comparisons revealed that gravity effects on error rates were specific to target sounds. Participants showed a significantly higher error rate in 0G compared to both 1G (p =.009, Cohen’s d = 0.81) and 1.8G (p =.028, Cohen’s d = 0.69), representing large and medium effect sizes, respectively. In contrast, no significant differences emerged across gravity conditions for the standard sounds (p >.05, $|d| \le 0.60$).

**Table 2:** Behavioural Performance like reaction time of the answers for the target sound for the oddball. Error Rate for the target and standard sound of the oddball. Displayed are means ± standard deviation.

	**Behavioural performance**		
	**Error rate [%]**		**Reaction time [ms]**
	Target sound	Standard sound	Target sound
0 G	$26.863 \pm 17.703$	$2.271 \pm 1.370$	$592.015 \pm 116.923$
1 G	$16.406 \pm 11.146$	$1.811 \pm 1.421$	$577.985 \pm 115.775$
1.8 G	$19.722 \pm 12.286$	$2.595 \pm 1.264$	$576.434 \pm 115.226$
Statistical test	Repeated measures ANOVA		Friedman test
p value	Interaction effect of *Gravity* and *Error Rate*:$p = .003$*		n.s ($\chi ^{2} = 4.333$,$p = .115$)
Post-hoc (t-test with Bonferroni correction)	**Target Sound:** 0 G vs. 1.8 G:$p = .028$* 0 G vs. 1 G:$p = .009$**		

**Fig. 8:**
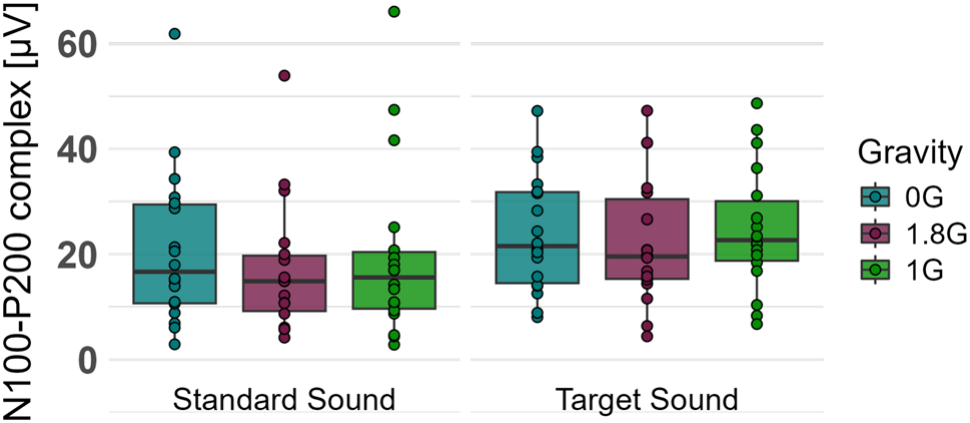
Amplitude of the N100-P200 complex, elicited by the standard and target sound of the oddball paradigm in 0G, 1.8G and 1G. Displayed are means ± standard deviation.

**Fig. 9:**
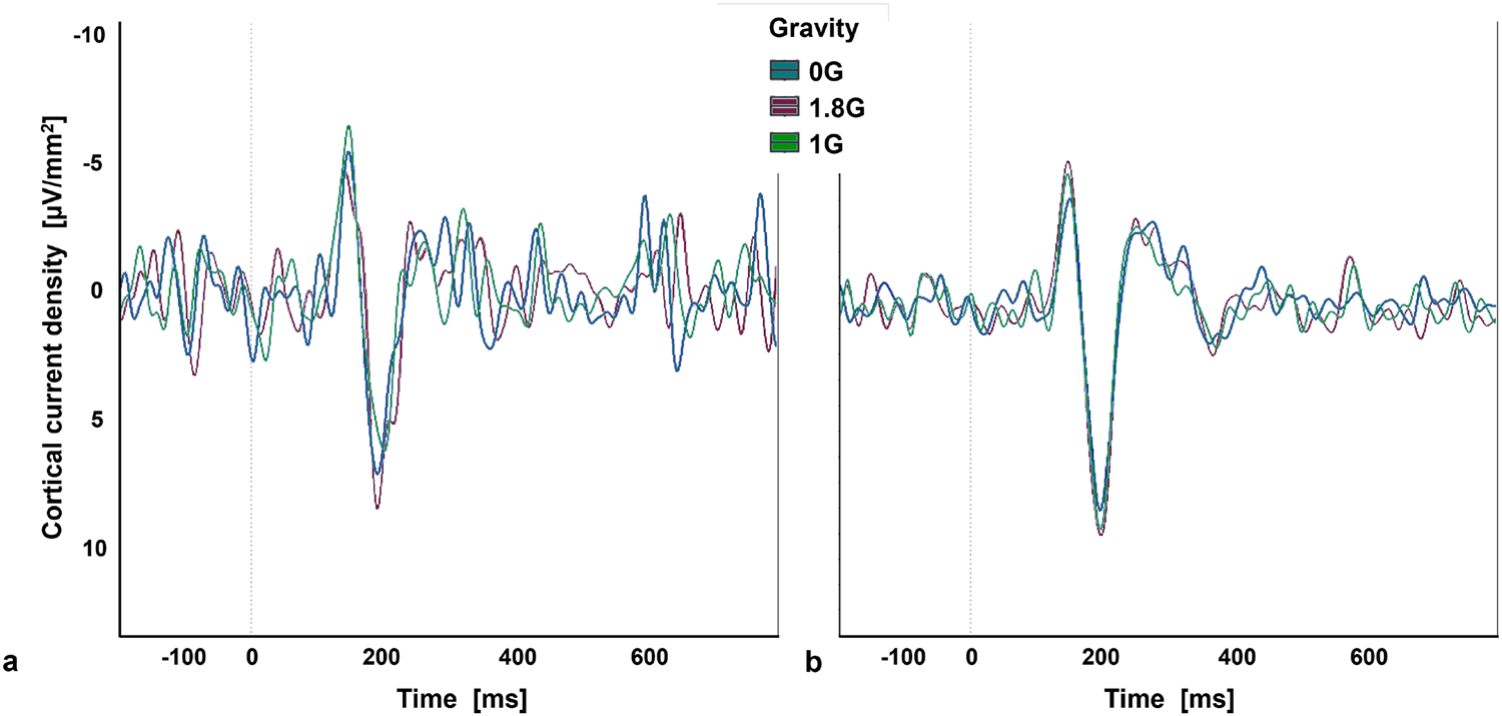
Averaged N100-P200 complex across electrode P7 after the occurrence of a target sound (**a**) and standard sound (**b**) of an auditory oddball paradigm.

The standard and target tones of the auditory oddball paradigm elicited a negative and a consecutive positive wave under the posterior electrode P7 and a negative wave over the fronto-central part, which was most pronounced under electrode C3. A two-way repeated measure ANOVA was conducted to evaluate the effect of different gravity levels on the processing of the standard and target sounds of the auditory oddball paradigm on the amplitude of the observed N100-P200 complex. The data was not normally distributed and therefore the ld.f2 model with the Wald-type (WTS) was calculated and showed no significant interaction effect of the processing of the auditory oddball paradigm and the gravity levels (Statistic: 3.543, p=.170, Figs. 8, 9, Table 3).

**Table 3:** Amplitude of the N100-P200 complex of event-related potentials for the oddball paradigm. Displayed are means ± standard deviation.

	Event-Related Potentials	
	N100-P200 complex	
	Standard Sound	Target Sound
	Amplitude [$\mu V$]	Amplitude [$\mu V$]
0 G	$20.843 \pm 14.642$	$23.924 \pm 11.193$
1 G	$19.535 \pm 16.540$	$24.727 \pm 11.713$
1.8 G	$16.966 \pm 12.390$	$22.316 \pm 12.066$
Statistical test	Ld.f2 model with Wald test	
p value	n.s	

**Fig. 10:**
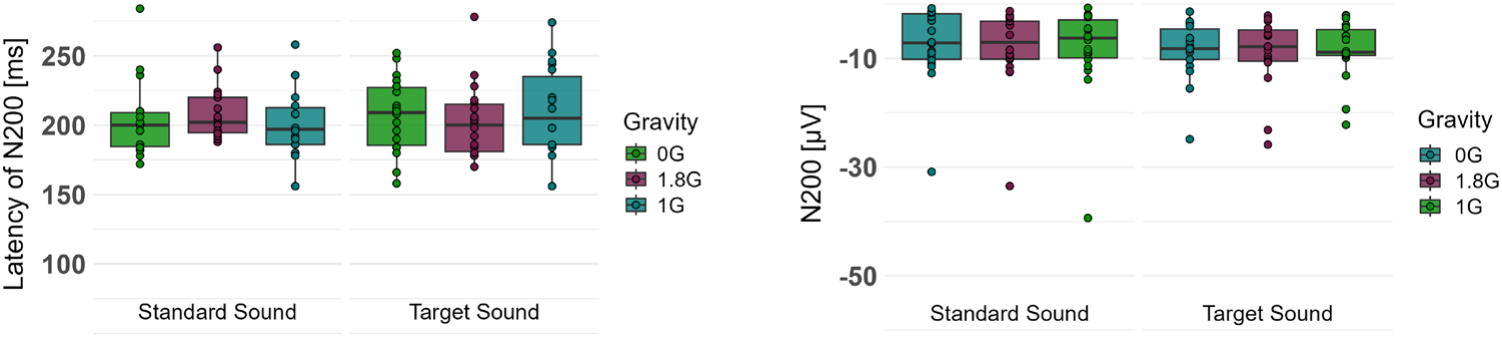
Latency (**a**) and Amplitude (**b**) of the negative event-related potential, elicited by the standard and target sound of the oddball paradigm in 0G, 1.8G and 1G. Displayed are means ± standard deviation.

**Fig. 11:**
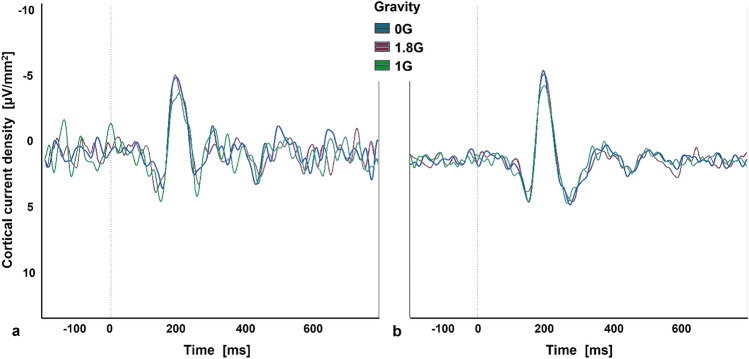
Averaged negative event-related potential across electrode C3 after the occurrence of a target sound (**a**) and standard sound (**b**) of an auditory oddball paradigm.

A two-way repeated measures ANOVA was performed to investigate the effects of different gravity levels on the processing of the standard and target sounds of the oddball paradigm in terms of latency and amplitude of the negative peak after approximately 200 ms. The data for the latency was not normally distributed and therefore the ld.f2 model with WTS was calculated and showed no significant interaction effect of the processing of the oddball paradigm and the gravity levels (Statistic: 3.299, p=.192). Regarding the amplitude of the negative peak, the data were not normally distributed, and the ld.f2 model with WTS showed no significant interaction effect of the processing of the oddball paradigm and the gravity levels (Statistic: 0.467, p=.792, Figs. 10, 11, Table 4).

## Discussion

This study investigates the effects of altered gravity levels on neurocognitive performance and electrocortical activity under three different gravity conditions in direct succession: 1 G (equivalent to Earth’s gravity), 1.8 G (hypergravity), and 0 G (weightlessness). For the first time, the focus is also on the task structure of the dual-task, as this might influence the allocation of cognitive resources. The analysis of electrocortical activity (LORETA) revealed no statistically significant differences between the gravity levels in all four brain lobes. This result is in contrast to previous studies that showed a significant reduction in brain activity in microgravity when two discrete tasks were combined in a dual-task^24^. However, the results are consistent with those from hypergravity and partial gravity, which showed no statistically significant reduction in brain activity in the frontal lobe^4,5^. At this point, it is questionable if LORETA data in microgravity are task-specific, depending on the cognitive demands involved. More intensive research of this parameter is required to establish how different task characteristics modulate electrocortical activity in altered gravity conditions.

The electrophysiological data from the current study demonstrated a pronounced negative peak after approximately 100 ms, followed by a positive deflection after around 200 ms, which is known as the N100-P200 complex and has been previously described by^43^, who observed it in conjunction with a cognitive task comprising a single discrete component^43^. This complex can be classified as an early event-related potential (ERP), which is closely associated with perceptual processing. In contrast, later ERPs, such as the P300 component, are associated with higher cognitive processes like stimulus evaluation^12,36^. Since an auditory oddball paradigm was used as a secondary task in this experiment, which had already triggered a clear P300 in previous studies with a discrete primary task, the absence of this component is noteworthy. The non-appearance may indicate that the continuous task employed in the current study was more demanding than the mental arithmetic task that had been previously investigated^4,5,24^. It is hypothesised that the continuous primary task in the study may have limited the cognitive processing of the different tones of the oddball paradigm, indicated by the absence of the P300 component.

Despite no gravity-dependent changes in reaction time could be observed, the error rate was found to be significantly higher in 0 G compared to 1.8 G and 1 G. These observations diverge from those of previous studies that employed a discrete primary task^4,5,41^. In these studies, the reaction time was observed to be shorter in microgravity, while the error rate remained unaltered in the reaction to the primary task. There was no change in the secondary task, which leads to the assumption of sufficient processing of the secondary task^42^. In contrast, no differences were observed in the behavioural data in a discrete primary task in hypergravity and partial gravity^4,5^.

The use of cognitive resources, known as cognitive workload, is based on two theories: the limited capacity theory^21^ and the multiple resource theory^38^. These theories emphasise that the human brain has a limited capacity to absorb and process information. They explain the relationship between the complexity of a task and performance, which decreases as the task becomes more complex. The absence of P300 detection in the study’s results may indicate elevated and persistent attention demands for the primary task. That could be further interpreted as an indication of a redistribution of available cognitive resources with the majority allocated to the Pac-Man game, and that the auditory stimuli of the oddball paradigm were no longer perceived in a differentiated manner. This hypothesize is supported by the fact, that no differences were observed regarding the performance of the primary task and the overall low number of collisions between Pac-Man and the ghosts. This suggests that participants performed at a consistent level throughout the experiment, regardless of the gravity level.

Prior research, including that of^3^, has demonstrated that dual-tasks typically necessitate a substantial allocation of cognitive resources and that the additional demand of a second task is difficult to quantify. It is frequently challenging to ascertain which element of the dual-task participants are concentrating on^3^. The reduced amplitude values of the event-related potentials observed in this study, compared to the results of^43^, the absence of a P300 component and the relatively stable performance of the primary task may be indicating, that the Pac-Man task being perceived as more complex and demanding than the discrete mental arithmetic task.

Another crucial neurophysiological parameter of this study is the observation of the N200 component, which may be interpreted as a Mismatch Negativity (MMN), as described earlier^2,30,31^in a dichotomic-listening study. The MMN is hypothesised to reflect an automatic neural mismatch process occurring between the input of a deviant stimulus and a sensory memory trace. Similarly^15^, distinguished the visual N2 from the auditory MMN, noting that the MMN responds to auditory deviants even when there is no focal attention to the stimuli^15^. It also appears to occur independently of task complexity and cognitive load^17,29^.

The combination of the results suggests that a continuous primary task in the context of a discrete secondary task requires a higher cognitive workload than a discrete primary task. This results in fewer resources being available for the secondary task. Understanding how the human brain allocates available cognitive resources, especially during dual-task situations, particularly in 0 G, is of major importance to astronauts and ground personnel who are responsible for prioritising the relevant tasks during space missions. For example, during the docking manoeuvre of the Soyuz capsule to the ISS, the astronauts must be able to manually dock the capsule to the ISS with six degrees of freedom and at the same time react to sounds from the spacecraft. On Earth, these findings can have implications for stressful situations, where two tasks have to be solved simultaneously, or when faced with a critical situation.

One limitation of the study was the fixed sequence of gravity levels ($1 G \rightarrow 1.8 G \rightarrow 0 G$), which was dictated by the physical constraints of a parabolic flight manouvre and could not be compensated for. Further, no demographic information were recorded in this study. However, since a within-subject design was applied, the influence of inter-individual differences were minimised and considered as less relevant to the primary research question.

Overall, the present study shows that examining ERPs in the context of dual-task performance can provide valuable insights into cognitive processing and workload management. The results highlight the need for further research into cognitive workload and attentional focus during dual-task performance in complex environments to improve overall performance and safety.

**Table 4:** Amplitude and Latency of the event-related potential N200 for the oddball paradigm. Displayed are means ± standard deviation.

Event-Related Potentials				
N200				
	Latency [ms]		Amplitude [$\mu V$]	
	Standard Sound	Target Sound	Standard Sound	Target Sound
0 G	$203.333 \pm 27.233$	$206.889 \pm 26.887$	$-7.502 \pm 7.125$	$-8.604 \pm 5.434$
1 G	$201.667 \pm 24.841$	$210.889 \pm 30.910$	$-8.300 \pm 8.688$	$-8.524 \pm 5.461$
1.8 G	$208.000 \pm 19.060$	$202.556 \pm 26.584$	$-8.085 \pm 7.442$	$-8.904 \pm 6.569$
Statistical test	Ld.f2 model with Wald test		Ld.f2 model with Wald test	
p-value	n.s.		n.s.	

## Data Availability

The datasets used and/or analysed during the current study available from the corresponding author on reasonable request.
